# Perioperative outcomes of non-intubated versus intubated video-assisted thoracoscopic surgery in different thoracic procedures: a propensity score-matched analysis

**DOI:** 10.1186/s12871-022-01667-9

**Published:** 2022-05-19

**Authors:** Chompunoot Pathonsamit, Apichat Tantraworasin, Sujaree Poopipatpab, Sira Laohathai

**Affiliations:** 1 Department of Anesthesiology, Faculty of Medicine Vajira Hospital, Navamindradhiraj University, Bangkok, Thailand; 2 Clinical Surgical Research Center, Department of Surgery, Faculty of Medicine, Chiang Mai University, Chiang Mai, Thailand; 3 Clinical Epidemiology and Clinical Statistic Center, Faculty of Medicine, Chiang Mai University, Chiang Mai, Thailand; 4 CardioThoracic Surgery Unit, Department of Surgery, Faculty of Medicine Vajira Hospital, Navamindradhiraj University, 681 Thanon Samsen, Khwaeng Wachira Phayaban, Khet Dusit, Krung Thep Maha Nakhon, Bangkok, Thailand

**Keywords:** Thoracic Surgery, Video-Assisted, Propensity Score, Non-intubated

## Abstract

**Background:**

Non-intubated video-assisted thoracoscopic surgery (NIVATS) is increasingly performed in different types of thoracic procedures. Based on the anesthetic perspective, the outcomes of this method are limited. General anesthesia with intubation and controlled ventilation for video-assisted thoracoscopic surgery (IVATS) is a standard technique. The current study aimed to compare the pulmonary gas exchange between NIVATS and IVATS, with a focus on desaturation event.

**Methods:**

This was a retrospective study conducted at Vajira Hospital. Data were collected from the hospital medical record database between January 9, 2019, and May 15, 2020. A propensity score-matched analysis was used to adjust the confounders by indications and contraindication between NIVATS and IVATS. The perioperative outcomes of VATS and NIVATS were compared by the regression analysis method.

**Results:**

In total, 180 patients were included in the analysis. There were 98 and 82 patients in the NIVATS and IVATS groups, respectively. After a propensity score matching, the number of patients with similar characteristics decreased to 52 per group. None of the patients in both groups experienced desaturation. The lowest oxygen saturation of the NIVATS and IVATS groups did not significantly differ (96.5% vs. 99%, respectively; *p* = 0.185). The NIVATS group had a significantly higher ETCO2 peak than the IVATS group (43 vs. 36 mmHg, respectively; *p* < 0.001). According to the regression analysis, the NIVATS group had a significantly shorter anesthetic induction time (Mean difference (MD) = -5.135 min (95% CI = (- 8.878)- (-1.391)) and lower volume of blood loss (MD = -75.565 ml (95%CI = (- 131.08)—(- 20.65) but a higher intraoperative ETCO2 than the IVATS group (MD = 4.561 mmHg (95%CI = 1.852—7.269). Four patients in the NIVATS group required conversion to intubation due to difficulties encountered when using the surgical technique (7.7%, *p* = 0.041). Seven patients in the IVATS group, but none in the NIVATS group, presented with sore throat (13.5% vs. 0%, respectively; *p* = 0.006). Moreover, none of the patients in both groups experienced postoperative pneumonia, underwent reoperation, or died.

**Conclusions:**

The anesthetic and surgical outcomes of NIVATS were comparable to those of IVATS.

## Background

General anesthesia with intubation and controlled ventilation for video-assisted thoracoscopic surgery (IVATS) is a standard technique. Several lung isolation devices such as double-lumen endobronchial tube (DLT), bronchial blocker, and single-lumen tube were applied to achieve a good surgical exposure. However, previous reports showed that the occurrence of complications correlated with intubation, which range from tracheal rupture [1–3] and dental damage [4] to sore throat. Moreover, ventilator-induced lung injury after one-lung ventilation (OLV) was reported in some studies [5].

Non-intubated video-assisted thoracoscopic surgery (NIVATS) is an alternative technique that has been increasingly performed during the last two decades. This technique is implemented for different types of thoracic procedures, such as wedge resection, segmentectomy, lobectomy, lung volume reduction, and mediastinal tumor surgery, with successful surgical outcomes [6–11].

According to the anesthetic perspective, studies about NIVATS technique reported in oxygenation and ventilation during procedure, complications correlated with anesthesia, and anesthetic and recovery time are limited. Some reports have shown that pulmonary gas exchange is preserved during the procedure. However, data about anesthetic-related complications are still lacking [12].

Therefore, the primary outcome of this study aimed to compare the pulmonary gas exchange between NIVATS and IVATS, with a focus on desaturation event, which was defined as an oxygen saturation (SpO2) of < 90% assessed using a pulse oximetry. The other outcomes were anesthetic induction and surgical time, conversion rate to IVATS, anesthetic and surgical complications, volume of blood loss, length of hospital stay, and 30-day mortality rate.

## Methods

This study was approved by the Vajira ethics committee (COA 103/63), and it was registered in the Thai Clinical Trials Registry (TCTR20201027004). The need for informed consent was waived due to the retrospective nature of the study.

### Study design and patients

In total, 180 VATS procedures in Vajira Hospital, Navamindradhiraj University, Thailand, were retrospectively analyzed. Data were collected from the hospital medical record database between January 9, 2019, and May 15, 2020.

The inclusion criteria were as follows: patients who were scheduled to undergo VATS for pulmonary resection and who were aged ≥ 18 years old with an American Society of Anesthesiologist physical status of I–III. The exclusion criteria in this study were as follows: patients who require reoperation, emergency surgery, and conversion to open thoracotomy. For selection criteria for non-intubate video assisted thoracoscopic surgery (NIVATS) was shown in Table 1.

**Table 1:** Contraindication criteria for Non-intubate Video assisted thoracoscopic surgery (NIVATS)

Contraindication for NIVATS patients
Difficult airway
Extensive pleural adhesion or previous pulmonary resection
Severe cardiopulmonary dysfunction or pulmonary hypertension
Persistent cough or chronic cough
Neurological conditions eg. Dementia, seizure, stroke
Morbid obesity (BMI > 30 kg/m2)
High intracranial pressure
High risk of regurgitation eg. Hiatal hernia
Resting hypoxaemia or hypercarbia
Coagulopathy
Contralateral phrenic nerve palsy
Complicate surgery eg. Sleeve lobectomy, Large tumor more than 7 cm, calcified lymph node
ASA Physical status classification > 3

### Sample size and statistical analysis

The sample size was calculated assuming that there was no difference in desaturation events between the two techniques. A previous study showed that 4% of desaturation events occurred in IVATS [13]. Approximately 48 patients were required per group, with an alpha value of 0.05 and beta value of 0.2. For data completion, 180 patients were included in the study.

All statistical analyses were conducted using Stata Statistical Software version SE 13 (StataCorp LP, College Station, TX, the USA). Categorical variables were presented as frequencies and proportions and continuous variables as median (P_25_–P_75_). A one-to-one propensity score-matching analysis was used to minimize the selection bias between the NIVATS and GAVATS groups. By Adjusted for differences in the baseline characteristics of patients. Logistic regression was used to calculate a propensity score, which evaluates confounding by indication and/or baseline covariates between the NIVATS and GAVATS groups. The variables included in the propensity score-matched model were age, sex, body mass index, smoking status, underlying disease, diagnosis, and thoracic procedure.. A Standardized differences (STD) between groups for all covariates were analyzed. STD value less than 0.2 refers to not statistically significant difference. The chi-square test was used to compare categorical variables and differences between the two groups. The Mann–Whitney U test was applied to assess continuous data and skewed distributed data. A linear regression analysis was performed to assess the associations between the anesthetic technique and anesthetic induction time, highest intraoperative end-tidal carbon dioxide (ETCO2), volume of blood loss, and length of hospital stay. A *p*-value of < 0.05 indicated a statistically significant difference.

### Anesthetic technique

All patients received premedication including paracetamol 500 mg and gabapentin 300 mg 2 h before surgery. Arterial line and Foley catheter were used in complicated surgeries alone such as large tumour ( size more than 6 cm), sleeve resection, tumour involve main pulmonary artery. Epidural or paravertebral blockade was not used in any cases.

### NIVATS group

The anesthetic technique used was based on the targeted controlled infusion of propofol and titration of fentanyl as total intravenous anesthesia. The depth of anesthesia was monitored using bispectral index to maintain the value between 40 and 60. Oxygen was supplemented via facemask with a reservoir bag with an oxygen flow of 10 L per min. Spontaneous ventilation was maintained during the procedures. Lung collapse was achieved via iatrogenic pneumothorax. For intraoperative hypoxemia prevention, we will perform positive airway pressure through facemask to resume bilateral lung ventilation and improved oxygenation when oxygenation saturation was under 90 percent. However, if patients are continued hypoxemia, we will consider intubation. At the end of surgery, lung re-expansion was performed by applying positive pressure via mask ventilation, and intravenous drugs were discontinued. Patients were transferred to the post-anesthetic care unit and then to the ward or intensive care unit according to their conditions.

### IVATS group

A double-lumen endobronchial tube (Broncho Cath DLT, Mallinckrodt Medical, Inc. St. Louis, MO) was inserted after induction with propofol, fentanyl, and cisatracurium. We selected a tube size of 32- or 35-Fr in women and 37- or 39-Fr in men. The proper tube position was confirmed based on breath sound and chest movement. Neuromuscular blockade was added according to schedule. Anesthesia was maintained by adjusting sevoflurane concentration and fentanyl titration properly according to hemodynamic changes. The protective ventilation strategy was used with a tidal volume of 4–5 mL/kg and a positive end-expiratory pressure of 5 cm H_2_O, with a peak pressure of < 30 cm H_2_O. A fractional inspired oxygen tension of 1.0 was applied during OLV. Lung collapse was achieved via DLT clamping. At the end of surgery. lung re-expansion was conducted via positive pressure ventilation using the tube. Patients were extubated at the end of surgery based on the criteria. Patients were transferred to the post-anesthetic care unit and then to the ward or intensive care unit according to their conditions.

### Surgical technique

The NIVATS and IVATS groups underwent surgery performed by a single thoracic surgeon. At the beginning of surgery, all patients received local infiltration and 3^th^–8^th^ intercostal nerve blockade with 2% xylocaine with 10 mL of adrenaline (1:200,000) combined with 20 mL of 0.25% bupivacaine under direct vision through camera in surgical field. Vagal nerve blockade, which aims to suppress cough reflex, was facilitated in the NIVATS group along using the same analgesic mixture. Patients routinely undergo chest tube placement at the end of surgery. In cautiously selected cases, tubeless surgery was considered based on the surgical condition of patients.

## Results

In total, 314 patients were included in the study. Among them, 134 were excluded, and 180 patients were finally included in the analysis. There were 98 and 82 patients in the NIVATS and IVATS groups, respectively. The two groups differed in terms of baseline characteristics, diagnosis, and procedure. The NIVATS group was younger and had a lower incidence of comorbidities than the IVATS group. Moreover, the NIVATS group frequently diagnosed with benign lesion and underwent wedge resection. After a propensity score matching, both groups were found to have similar characteristics including age, body mass index, sex, smoking status, comorbidities, diagnosis, and thoracic procedure. The number of patients decreased to 52 per group (Table 2).

**Table 2:** Patient characteristics before and after propensity score matching according to anesthesia technique

Variables	Before Propensity Score Matching				After Propensity Score Matching			
	**(Full Patient Cohort)**				**(Propensity Score Matched Patient Cohort)**			
	**NIVATS** **(*n* = 98)**	**IVATS** **(*n* = 82)**	***P*-value**	**STD**	**NIVATS** **(*n* = 52)**	**IVATS** **(*n* = 52)**	***P*-value**	**STD**
**Age (years); median (P**_**25**_**—P**_**75**_**)**	54 (38.8—65)	60 (44.8—69.3)	0.034*	-0.309	55 (43.5—65.5)	59.5 (40.5—68)	0.492	-0.129
**Gender; n (%)**			0.663	0.065			0.693	0.078
Male	47 (48%)	42 (51.2%)			22 (42.3%)	24 (46.2%)		
Female	51 (52%)	40 (48.8%)			30 (57.7%)	28 (53.9%)		
**BMI (kg/m**^**2**^**); median (P**_**25**_**—P**_**75**_**)**	22.1 (19.6—24.7)	22.8 (20.2—26.7)	0.179	-0.212	22.6 (19.8—24.9)	22.2 (18.7—26.5)	0.818	0.024
**Smoking status; n (%)**			0.721	0.053			0.426	0.156
Non-smoker	78 (79.6%)	67 (81.7%)			45 (86.5%)	42 (80.8%)		
Smoker	20 (20.4%)	15 (18.3%)			7 (13.5%)	10 (19.2%)		
**Underlying disease; n (%)**								
Diabetes mellitus	22 (22.4%)	19 (23.2%)	0.908	-0.017	13 (25%)	11 (21.2%)	0.642	0.039
Hypertension	30 (30.6%)	39 (47.6%)	0.020*	-0.351	20 (38.5%)	21 (40.4%)	0.841	0.000
Dyslipidemia	21 (21.4%)	28 (34.1%)	0.056	-0.285	12 (23.1%)	12 (23.1%)	1.000	0.000
Cardiovascular disease	0 (0%)	5 (6.1%)	0.018*	-0.358	0 (0%)	0 (0%)		
Cerebrovascular disease	1 (1%)	5 (6.1%)	0.094	-0.275	1 (1.9%)	1 (1.9%)	1.000	0.000
COPD	3 (3.1%)	9 (11%)	0.040*	-0.312	3 (5.8%)	2 (3.9%)	0.647	0.090
**Diagnosis; n (%)**			0.022*	0.349				
Malignant	61 (62.2%)	64 (78.1%)			36 (69.2%)	37 (71.2%)	0.830	0.042
Benign	37 (37.7%)	18 (21.9%)			16 (30.8%)	15 (28.9%)	0.596	
**Procedure; n (%)**			0.000*	0.600			0.596	0.200
Wedge	78 (79.6%)	43 (52.4%)			37 (71.2%)	37 (71.2%)		
Segmentectomy	2 (2%)	5 (6.1%)			1 (1.9%)	0 (0%)		
Lobectomy	18 (18.4%)	34 (41.5%)			14 (27%)	15 (27.9%)		

Abbreviation: *STD* Standardized difference; NIVATS, Non-intubated video-assisted thoracoscopic surgery; IVATS, General anesthesia with intubation and controlled ventilation for video-assisted thoracoscopic surgery; BMI, body mass index; COPD, chronic obstructive pulmonary disease

In terms of anesthetic outcomes, both groups did not present with desaturation. The lowest SpO2 between the two groups did not significantly differ (96.5% vs. 99%, *p* = 0.185). By contrast, the NIVATS group had a significantly higher ETCO2 peak than the IVATS group (43 vs. 36 mmHg, *p* < 0.001) (Table 3). The ETCO2 of NIVATS group was significantly higher than that of IVATS group. The anesthetic induction time of the NIVATS group was significantly shorter than that of the IVATS group (10 vs. 15 min, *p* = 0.005) (Table 3). There was no difference in the overall anesthetic induction and operative time between the two groups.

**Table 3:** Peri-operative outcomes between two anesthesia technique

Variables	Before Propensity Score Matching				After Propensity Score Matching			
	**(Full Patient Cohort)**				**(Propensity Score Matched Patient Cohort)**			
	**NIVATS** **(*n* = 98)**	**IVATS** **(*n* = 82)**	***P*-value**	**STD**	**NIVATS** **(*n* = 52)**	**IVATS** **(*n* = 52)**	***P*-value**	**STD**
**Anesthetic induction time (min);** **median (P**_**25**_**—P**_**75**_**)**	10 (5—15)	13.5 (10—20)	0.009*	-0.318	10 (5—15)	15 (10—20)	0.005*	-0.534
**Overall anesthetic time (min);** **median (P**_**25**_**—P**_**75**_**)**	125 (90—201.3)	122.5 (95—171.3)	0.725	0.083	145 (97.5—210)	127.5 (105—175)	0.651	0.121
**Operative time (min);** **median (P**_**25**_**—P**_**75**_**)**	60 (40—95)	112.5 (63.8—171.3)	0.000*	-0.701	67.5 (40—107.5)	80 (55—167.5)	0.059	-0.368
**Anesthetic results**								
Prevalence of desaturation	0 (0%)	0 (0%)			0 (0%)	0 (0%)		
The lowest intraoperative SpO_2_ (%); median (P_25_—P_75_)	100 (96.8—100)	99.5 (95.8—100)	0.056	0.097	96.5 (92—100)	99 (97—100)	0.185	-0.056
The highest intraoperative ETCO2 (mmHg); median (P_25_—P_75_)	44 (40—48)	36.5 (34—40)	0.000*	0.837	43 (40.5—48)	36 (34—41)	0.000*	0.655
**Surgical results**								
Blood loss (ml); median (P_25_—P_75_)	20 (10—50)	50 (10—150)	0.000*	-0.531	20 (10—50)	50 (10—125)	0.008*	-0.534
Blood transfusion; n (%)	1 (1%)	4 (4.9%)	0.179	0.23	0 (0%)	3 (5.8%)	0.079	0.350
Tubeless surgery; median (P_25_—P_75_)	3 (3—5)	5 (4—8.3)	0.000*	-0.543	3 (3—4)	5 (4 -7)	< 0.001*	-0.408
**Complications**								
Conversion to GAVATS; n (%)	6 (6.1%)	0 (0%)	0.032*	0.361	4 (7.7%)	0 (0%)	0.041*	0.408
Sore throat; n (%)	0 (0%)	10 (12.2%)	0.000*	0.527	0 (0%)	7 (13.5%)	0.006*	0.558
Other anesthetic complication; n (%)	4 (4.1%)	0 (0%)	0.127	0.292	2 (3.9%)	0 (0%)	0.153	0.283
Reoperation; n (%)	2 (2%)	1 (1.2%)	1.000	0.065	0 (0%)	0 (0%)		
Pneumonia; n (%)	0 (0%)	0 (0%)			0 (0%)	0 (0%)		
**Chest radiograph results;** n (%)			0.749	0.047			0.169	0.270
Normal	92 (93.9%)	76 (92.7%)			51 (98.1%)	48 (92.3%)		
Atelectasis	6 (6.1%)	6 (6.1%)			1 (1.92%)	4 (7.7%)		
**Hospital stays** (day**);** median (P_25_—P_75_)	5 (4—7.3)	7 (5—11)	0.000*	-0.426	5 (4—7)	6 (5—10)	0.004*	-0.341
**Mortality;** n (%)	0 (0%)	0 (0%)			0 (0%)	0 (0%)		

Abbreviation: *STD* Standardized Difference;, *NIVATS* Non-intubated Video-assisted Thoracoscopic Surgery, IVATS, General anesthesia with intubation and controlled ventilation for video-assisted thoracoscopic surgery, *SpO*_*2*_ Oxygen Saturation, *ETCO2* End-tidal Carbon Dioxide

In terms of surgical outcomes, the NIVATS group had a significantly lower volume of blood loss and a shorter length of hospital stay than the IVATS group (20 vs. 50 mL, *p* = 0.008 and 5 vs. 6 days, *p* = 0.004). There was no mortality in both groups.

In the regression analysis, the NIVATS group had a significantly shorter anesthetic induction time and lower volume of blood loss but a higher intraoperative ETCO2 than the IVATS group (Table 4).

**Table 4:** Multiple linear regression analysis of each outcome variable comparing between NIVATS and IVATS

Outcome variables	Mean difference	95% CI	*P*—value
Anesthetic induction time (min);	-5.135	(- 8.878)- (-1.391)	0.008*^a^
The highest intraoperative ETCO2 (mmHg);	4.561	1.852—7.269	0.001*^b^
Estimate blood loss (ml);	-75.565	(- 131.08)—(- 20.65)	0.008*^c^
Hospital stays (day);	-1.923	(- 3.912)—0.027	0.053^d^

Analyzed by Multiple Linear Regression model

^a^ Adjusted by Age, BMI, Gender, Smoking status and Diagnosis

^b^ Adjusted by Age, Gender, DM, HT, Stroke and operation time

^c^ Adjusted by Age, Gender, BMI and Procedure

^d^ Adjusted by Age, Gender, Procedure, Conversion to GAVATS, Sore throat and Other anesthetic complication

In terms of postoperative complications, four patients in the NIVATS group required conversion to intubation with controlled ventilation during surgery (7.7%, *p* = 0.041). The reasons for conversion were difficulties using the surgical technique due to factors such as severe calcified lymph node and bleeding. In total, seven patients in the IVATS group, but none in the NIVATS group, experienced sore throat (13.5% vs. 0%, *p* = 0.006). There was no difference in terms of other anesthetic complications such as nausea and vomiting between the two groups. None of the patients had postoperative pneumonia or reoperation. There was no difference in terms of atelectasis on postoperative chest radiography between the two groups.

## Discussion

Based on the anesthetic perspective, hypoxia and hypercapnia are the most common issues during OLV. Intubation with controlled ventilation is an essential strategy. Hence, this study aimed to compare the pulmonary gas exchange and perioperative conditions between NIVATS and IVATS for thoracic surgery. Results showed that the NIVATS and IVATS groups did not experience desaturation. In previous studies, the incidence of desaturation during OLV via the tube was between 4 and 20% [13–15].

The high incidence rate might be attributed to different factors, including varying characteristics of patients, anesthetic technique and expertise, surgical technique, and procedure. In addition, the difference in the cutoff value for desaturation could be one of the reasons. In our institution, we commonly used 100% oxygen during OLV in both anesthetic techniques. However, the NIVATS group had a slightly lower intraoperative SpO2 than the IVATS group, without desaturation event. This finding indicated that oxygen administration via facemask can prevent hypoxia in NIVATS. Moreover, oxygen toxicity should be a cause of concern. Patients with lung cancer after chemotherapy and exposure to high oxygen concentrations may increase the risk of reactive oxygen species-mediated lung injury [16]. In addition, a previous report showed a greater degree of lung injury after 3 h of OLV in hyperoxic animals [17].

Hypercapnia cannot be prevented in prolonged NIVATS. The main cause is paradoxical respiration and hypoventilation from collapsed non-dependent lung. However, Paco_2_ was more likely to decrease and return to normal immediately after surgery [18]. The peak ETCO2 of the NIVATS group was significantly higher than that of the IVATS group even after the regression analysis. This finding was similar with that of other reports [11, 12]. The effect of brain metabolism and function in hypercapnia state was controversial, and some studies showed that acute hypercapnia increases intracranial pressure, decreases cerebral perfusion, and precipitates cerebral ischemia [19, 20]. Another study showed that hypercapnia caused reduced metabolism and spontaneous neural activity in the brain, thereby resulting in a lower arousal state [21]. By contrast, several studies revealed that hypercapnia had a neuroprotective effect. Cheng and coworkers assessed patients who experienced hypercapnia during bronchoscopic intervention, and results showed that mild to moderate hypercapnia promoted cognitive activity [22]. In addition, permissive hypercapnia is not likely to affect the performances of patients [23].

Other results showed that the overall anesthetic induction and operative times did not differ between the NIVATS and IVATS groups. However, the values in this study were still lower than those in others [12, 24]. These findings might be attributed to the non-routine use of fiberoptic bronchoscopy to validate DLT position and the difference in surgical procedure.

Sore throat is a cause of concern as it affects patient satisfaction after anesthesia. A previous study showed that about 10%–43.3% of patients who were intubated using the double-lumen tube up to 24 h after surgery presented with sore throat [25]. Another research revealed that the incidence of sore throat was high at 57.5% even in single-lumen intubation [26]. Our study showed that such an event was significantly correlated with the intubation technique.

In the era of Enhance Recovery After Surgery (ERAS) program, the non-intubated technique could promote ERAS program based on our study. The NIVATS group had a lower volume of intraoperative blood loss and a shorter length of hospital stay than the IVATS group. Moreover, none of the patients in the NIVATS group presented with sore throat. In terms of other anesthetic complications, the NIVATS group had a higher proportion of patients who experienced nausea and vomiting caused by anesthesia than the IVATS group. However, the results did not significantly differ. Thus, prophylactic treatment for postoperative nausea and vomiting should be included in the standard protocol.

In our study, the incidence of atelectasis confirmed on chest radiography was low. However, this finding was in contrast with that of the study of Lan and coworkers, and the difference may be associated with the type of surgery. The current research included different types of pulmonary resection procedures, and other studies focused on lobectomy alone.

Currently, NIVATS is used in our institution, and it is applied in several types of VATS procedures [27–29]. Therefore, this study could show the benefit of NIVATS particularly in terms of anesthetic perspective because all procedures were performed by a single surgeon with a similar technique.

The current study had several limitations. First, this was a retrospective study. Thus, selection bias could not be totally ruled out although the propensity score matching was used to decrease the selection bias or confounders by indication/contraindication. Second, SpO2 and ETCO2 were not the best parameters that can represent pulmonary gas exchange during OLV. However, noninvasive continuous monitoring was practical during anesthesia, and arterial blood gas analysis might be appropriate in such a case. Third, hemodynamic parameters including time to recovery from anesthesia, pain score, and cognitive function were not considered. Nevertheless, further prospective clinical trials should be conducted to address these limitations.

## Conclusions

IVATS and NIVATS had comparable anesthetic and surgical outcomes. Hence, NIVATS can be alternative technique as it is safe for some patients.

## Data Availability

The datasets analyzed during the current study are not publicly available due to unavailable to give consent to all participates but are available from the corresponding author on reasonable request.

## Abbreviations

NIVATS: Non-intubated video-assisted thoracoscopic surgery
IVATS: Intubation and controlled ventilation for video-assisted thoracoscopic surgery
OLV: One-lung ventilation
ETCO2: End-tidal carbon dioxide
DLT: Double-lumen endobronchial tube
SpO2: Oxygen saturation
